# Objective pathogen monitoring in nursery and finisher pigs by monthly laboratory diagnostic testing

**DOI:** 10.1186/s40813-020-00161-3

**Published:** 2020-09-08

**Authors:** Nicole B. Goecke, Maja Kobberø, Thomas K. Kusk, Charlotte K. Hjulsager, Ken Steen Pedersen, Charlotte S. Kristensen, Lars E. Larsen

**Affiliations:** 1 Centre for Diagnostics, Technical University of Denmark, 2800 Kgs Lyngby, Denmark; 2 Present address: University of Copenhagen, 1870 Frederiksberg C, Denmark; 3 Present address: Statens Serum Institut, 2300 Copenhagen S, Denmark; 4 Ø-Vet A/S, 4700 Næstved, Denmark; 5 SEGES Danish Pig Research Centre, 1609 Copenhagen V, Denmark

**Keywords:** Diagnostics, Monitoring, High-throughput real-time PCR, Coughing, Diarrhoea, Respiratory viruses, Respiratory bacteria, Enteric viruses, Enteric bacteria

## Abstract

**Background:**

Infectious diseases are of great economic importance in commercial pig production, causing both clinical and subclinical disease, with influence on welfare, productivity, and antibiotic use. The causes of these diseases are often multifactorial and laboratory diagnostics are seldom routinely performed. The aim of the present study was to explore the benefits of monthly pathogen monitoring in nursery and finisher herds and to examine association between laboratory results and observed clinical signs, including coughing and diarrhoea. Three monthly samplings were conducted in three different age groups in six nursery and four finisher production units. For each unit, two pens were randomly selected in each age group and evaluated for coughing and diarrhoea events. Furthermore, faecal sock and oral fluid samples were collected in the selected pens and analysed for 18 respiratory and enteric viral and bacterial pathogens using the high-throughput real-time PCR BioMark HD platform (Fluidigm, South San Francisco, USA).

**Results:**

In total, 174 pens were sampled in which eight coughing events and 77 diarrhoeic events were observed. The overall findings showed that swine influenza A virus, porcine circovirus 2, porcine cytomegalovirus, *Brachyspira pilosicoli*, *Lawsonia intracellularis*, *Escherichia coli* fimbria types F4 and F18 were found to be prevalent in several of the herds. Association between coughing events and the presence of swine influenza A virus, porcine cytomegalovirus (Cq ≤ 20) or a combination of these were found. Furthermore, an association between diarrhoeic events and the presence of *L. intracellularis* (Cq ≤ 24) or *B. pilosicoli* (Cq ≤ 26) was found.

**Conclusions:**

The use of high-throughput real-time PCR analysis for continuous monitoring of pathogens and thereby dynamics of infections in a pig herd, provided the veterinarian and farmer with an objective knowledge on the distribution of pathogens in the herd. In addition, the use of a high-throughput method in combination with information about clinical signs, productivity, health status and antibiotic consumption, presents a new and innovative way of diagnosing and monitoring pig herds and even to a lower cost than the traditional method.

## Background

Respiratory and intestinal infections are of major importance in commercial pig production resulting in reduced productivity, impaired animal welfare, increased mortality and increased consumption of antibiotics. The cause of these diseases are often multifactorial and the prevalence and combination of pathogens can fluctuate over time due to e.g. changes in management, environment, season or stage of production [1–5].

Respiratory diseases are one of the major problems in modern pig production worldwide and are often referred to as Porcine Respiratory Disease Complex (PRDC), which is a polymicrobial infection caused by a combination of various primary and secondary respiratory viral and bacterial pathogens. Environmental conditions, management factors, population size and factors such as age and genetics also play roles in the outcome of PRDC [1, 6]. Agents associated with PRDC include porcine circovirus type 2 (PCV2), porcine cytomegalovirus (PCMV), porcine reproductive and respiratory syndrome viruses 1 and 2 (PRRSV-1, − 2), swine influenza A virus (swIAV), *Actinobacillus pleuropneumoniae*, *Bordetella bronchiseptica*, *Glaesserella parasuis*, *Mycoplasma hyopneumoniae*, *Mycoplasma hyorhinis*, *Pasteurella multocida* and *Streptococcus suis*. Porcine Respiratory Disease Complex is most commonly observed in growing and finishing pigs, with mortality rates ranging from 2 to 10% and morbidity rates ranging from 10 to 40%. The clinical picture of PRDC is characterized by coughing, fever, dyspnoea, decreased feed intake and even fatal pneumonia [1, 6–9].

Pathogens involved in respiratory disease in pigs vary among countries, herds and production sites, making general treatment and control regimes for PRDC difficult to develop [8, 10]. Due to the polymicrobial nature of the disease, a range of different diagnostic samples and techniques may be employed in the investigation of a single case, including nucleic acid, antigen and antibody detection. For this, oral fluid samples are ideal for diagnosing PRDC due to the residency of the pathogens in the respiratory tract [3]. The use of oral fluid as a sampling method is a relatively new diagnostic method used for detection of pathogens. This sampling method has the advantage that it is practical, minimizes stress on the animals, offers the possibility of testing a large number of individuals in an aggregate sample and is economically beneficial compared to nasal swab and blood samples collection [3, 11, 12]. On the other hand, analysing oral fluid samples can be challenging due to the risk of contamination from faeces, nasal secretions or from the environment. Furthermore, isolating RNA from these biological samples can be difficult as it is easily degraded. Therefore, pre-processing and storage conditions such as time and temperature can be critical in the analysis of oral fluid samples [3, 11].

Intestinal diseases are another critical factor in modern pig production. The multifactorial disease “post-weaning diarrhoea” is associated with dehydration, reduced feed intake and thereby reduced growth and increased mortality [5]. Intestinal diseases in pigs can be caused by a wide range of viral and bacterial pathogens. The most frequently detected pathogens associated with intestinal disease in nursery pigs are *Lawsonia intracellularis*, *Brachyspira pilosicoli*, *Escherichia coli* fimbria types F4 and F18, whereas for finisher pigs especially *L. intracellularis* and *B. pilosicoli* are involved in enteric disease [13, 14]. The viruses; rotavirus A and PCV2 can also cause enteric disease. Rotavirus A is a known diarrhoea causing agent in pigs and has been associated with acute gastroenteritis, usually seen in young animals [15, 16]. Porcine circovirus type 2 has not been proven to be a primary cause of diarrhoea in pigs, however, systemic PCV2 may indirectly contribute to enteric diseases due to its immunosuppressive effect [17–19]. Coronaviruses such as transmissible gastroenteritis virus, porcine deltacoronavirus and porcine epidemic diarrhoea virus can also induce enteric diseases, but Denmark and many other European countries are free of these viruses [20].

The use of laboratory diagnostics in Danish pig production is limited and is often carried out only once a year, which is a requirement before batch medication can be used according to Danish legislation. The results of the diagnostic tests are often of limited value and therefore, prophylactic and therapeutic interventions are often initiated based solely on clinical signs [21]. The major reason for the limited use of laboratory diagnostics is that the traditional methods are expensive and resource demanding. To overcome these limitations, we have developed a high-throughput real-time PCR (rtPCR) detection system using the BioMark HD platform (Fluidigm, South San Francisco, USA), which is capable of detecting 18 significant porcine viruses and bacteria in the same setup [22].

The aim of the present study was to investigate the pathogen patterns in ten Danish production units over a 3 months period (September–November) and compare the findings with the observed clinical signs of coughing and diarrhoea in order to create a more objective basis for intervention. Furthermore, the study aimed to investigate the prevalence of different pathogens and their relation to clinical disease with a special focus on pathogens involved in PRDC and enteric diseases in pigs after weaning.

## Results

In total, 174 pens in ten production units were included in the study. In addition to the clinical registrations of coughing and diarrhoea, 172 oral fluid and 174 faecal sock samples were collected. It was not possible to collect oral fluid samples from the youngest pigs in November in herd 2 (2 N) and therefore, the total number of oral fluid samples was 172 instead of 174. The clinical registrations recorded eight coughing events (4.7% of the pens) during the 3 months of sampling, and the events were only observed in the nursery pigs. Furthermore, 77 (44.3%) of the collected pens were defined as pens with diarrhoeic events, of which the prevalence of diarrhoeic events was 38.0% for the nurseries and 54.5% for the finishers. In all production units, the rtPCR results were consistent with the specific pathogen free (SPF) [23] status of the herds (Table 1), including no detection of PRRSV-1 and -2 in any of the production units.

**Table 1:** Information and vaccination strategies for each of the production units. N: nursery, F: finisher

	1N	1F	2N	3N	3F	4N	4F	5N	6N	6F
**SPF status** [23]	Unknown	Unknown	Blue + AP12	Blue	Blue	Blue + M. hyo	Blue + M. hyo	Blue + M. hyo	Blue + M. hyo+ AP12	Blue + M. hyo+ AP12
**swIAV status**	Not diagnosed	Not diagnosed	Positive	Positive	Positive	Positive	Positive	Positive	Positive	Positive
**Sows per year**	400	400	770	560	560	-	2500	2500	735	730
**No. of pen units**	1800	600	3000	1500 + 1400	?	2020	2880	2500	?	1700
**No. of produced 30 kg pigs per year**	13500	-	33000	19000 (sell 5500 per year)	-	20000	-	80000	23500	-
**No. of produced finishing pigs per year**	-	4000	-	-	13500	-	12300	-	-	6000
**Type of farm**	Farrow-to-finisher	Farrow-to-finisher	Farrow-to-finisher	Farrow-to-finisher	Farrow-to-finisher	Nursery and finishers	Nursery and finishers	Sows and nursery	Farrow-to-finisher	Farrow-to-finisher
**No. of geographical sites**	1 nursery site (total 1)	1 finisher site (total 1)	2 nursery sites (total 7)	2 nursery sites (total 3)	2 finisher sites (total 3)	1 nursery site (total 3)	3 finisher sites (total 3)	2 nursery sites (total 3)	1 nursery site (total 1)	1 finisher site (total 1)
**Production**	Weekly	Weekly	Weekly	Every 14^th^ day	Every 14^th^ day	Every 14^th^ day	Every 14^th^ day	Weekly	Weekly	Weekly
**Daily weight gain, g**	459	1026	486	485 (site 1) 440 (site 2)	976 (site 1) 956 (site 2)	473	932	385	489	1022
**% of dead pigs per pigs produced**	1.3	2.9	1.8	2.2 (site 1) 2 (site 2)	1.6 (site 1) 2.4 (site 2)	1.6	1.7	4	1.8	4.5
**Vaccination status**										
***Erysipelothrix rhusiopathiae***	+	+								
***Erysipelothrix rhusiopathiae*+ parvovirus**	+	+	+	+	+			+	+	+
**Glässers disease (*G. parasuis*)**	+	+								
**swIAV**	+	+		+	+			+	+	+
**A(H1N1)pdm09**			+						+	+
***M. hyopneumoniae***			+			+	+		+	+
**PCV2**				+	+			+		
***E. coli*+*C. perfringens***	+	+	+	+	+			+	+	+
***E. coli*F4/F18**			+	+	+					

### Individual herd analysis

#### Herd 1: nursery (1 N) and finisher (1F) pigs

In herd 1, three age groups were sampled in the nurseries, whereas only two age groups were sampled in the finishers due to continuous flow and no clear separation of age groups. In the early nursery period, coughing episodes were often observed, but a coughing event was only recorded in one pen (Additional file 1A). In the affected pen, swIAV, PCMV and *A. pleuropneumoniae* were detected in the oral fluid sample. Swine influenza A virus was detected in all age groups in the nurseries, while it was only found sporadically in the finishers. *Actinobacillus pleuropneumoniae* was found in all pens in October and November in the finishers, while it was only found in single pens in the other samplings. Porcine cytomegalovirus and *S. suis* type 2 were found to circulate in both nurseries and finishers in all pens. Furthermore, *M. hyorhinis* was detected in almost all pens. In general, the piglets’ weight at weaning were below the optimal in this herd. Seventeen diarrhoeic events (Additional file 1A and 1B) were detected and distributed between the nurseries and finishers, although clinical diarrhoea was only observed in few pens after weaning. The analysis of the faecal sock samples showed that *E. coli* F4 and *E. coli* F18 were detected in the beginning of the nursery period in all 3 months, while *L. intracellularis* was detected at the end of the nursery period. Rotavirus A was also present in several of the nursery pens. In the finisher pens, clinical diarrhoea was observed in almost all pens although intestinal pathogens were only detected in few of them.

#### Herd 2: nursery pigs (2 N)

It was not possible to collect oral fluid samples from the youngest pigs in November due to their lack of interests in chewing in the rope. Three coughing events (Additional file 1C) were registered in this herd, all in the early nursery period in September and October. In these pens, swIAV and PCMV were detected with quantification cycle (Cq) values below 15. *Streptococcus suis* type 2 was circulating in all pens, while PCMV, PCV2, PCV3, *M. hyorhinis* and *A. pleuropneumoniae* were detected in many of the pens. Furthermore, four diarrhoeic events (Additional file 1C) were detected in herd 2 and *L. intracellularis* was found in all four affected pens in the faecal sock samples. Rotavirus A was found in nearly all pens, whereas *E. coli* F4 and *E. coli* F18 were found to be most prevalent in the beginning of the nursery period. *Lawsonia intracellularis* was found in the mid and late nursery period with the lowest Cq values in November.

#### Herd 3: nursery (3 N) and finisher (3F) pigs

Coughing events were detected in two pens in the nurseries (Additional file 1D). The analysis of the oral fluid samples showed that PCMV was detected in nearly all pens, but the virus was found with the lowest Cq values in the clinical affected pens. Swine influenza A virus was detected in one of the clinical affected pens and was also detected in the beginning of the nursery period in September and October, whereas in November it was detected in the middle to late nursery period. In contrast, swIAV was only detected sporadically in the finishers. Porcine circovirus type 3, *M. hyorhinis* and *S. suis* type 2 were also detected in many of the pens. Four diarrhoeic events (Additional file 1D) were registered in the nursery pens and eight diarrhoeic events (Additional file 1E) were registered in the finishers. No clear pattern was observed for the findings of *B. pilosicoli*, *L. intracellularis*, *E. coli* F4 and *E. coli* F18 in the faecal sock samples, since all these bacteria were detected sporadically in all age groups.

#### Herd 4: nursery (4 N) and finisher (4F) pigs

Clinically, no coughing events were observed in this herd during the three sampling months. This is in line with the finding that swIAV was only detected in the oral fluid samples from two pens in this herd. Between the samplings in October and November, the veterinarians observed clinical signs of wasting and uneven weight distribution 15–25 days after arrival to the nursery. Multiple factors could be the reason for this. Porcine circovirus type 2 was found in all age groups and with Cq values below 16 in several of the finisher pens, therefore, this virus could potentially have had an impact on productivity and secondary infections. Furthermore, *M. hyorhrinis*, *B. bronchiseptica* and PCV3 were found sporadically in the nurseries and finishers. Fifteen diarrhoeic events were observed (Additional file 1F and 1G). *Escherichia coli* F4 and\or *E. coli* F18 was detected in all of the faecal sock samples from the early nursery period. *Brachyspira pilosicoli*, sometimes in combination with *L. intracellularis*, dominated in the mid to late nursery period. Infections with *B. pilosicoli* and *L. intracellularis* seemed to extend to the finishing period. In November, all pens in the nurseries were positive for diarrhoeic events, which coincided with detection of *E. coli* F4 and *E. coli* F18 20 days after weaning. In addition, findings of *B. pilosicoli* and *L. intracellularis* 34 and 48 days after weaning could also explain the symptoms observed. Rotavirus A was primarily detected in September and October in the nurseries.

#### Herd 5: nursery pigs (5 N)

One coughing event was detected in this herd (Additional file 1H) and the affected pen was tested positive for swIAV and PCMV in the oral fluid sample. Furthermore, coughing was noticed with high frequency in the youngest age group in all 3 months, even though it was not correlated to coughing events. In general, swIAV was detected with the lowest Cq values at 12–15 days after weaning and with the highest Cq values in the late nursery period in September and October. Porcine cytomegalovirus, *S. suis* type 2 and *M. hyorhrinis* were detected in all pens, while PCV2 was detected in almost all pens. Several pens were also positive for PCV3 and *B. bronchiseptica*. Diarrhoea was rarely observed and only one diarrhoeic event was recorded (Additional file 1H). In the affected pen, *B. pilosicoli* was detected with a Cq value of 13 in the faecal sock sample. *Lawsonia intracellularis* and rotavirus A were also present but with Cq values above 23. Rotavirus A was found in almost all pens, while *L. intracellularis* only was found in the late nursery period in all three samplings. *Escherichia coli* F4 and *E. coli* F18 were found mainly in the early to mid nursery period.

#### Herd 6: nursery (6 N) and finisher (6F) pigs

One coughing event was noticed in this herd in the mid nursery period in November (Additional file 1I). In the affected pen, PCMV and *A. pleuropneumoniae* were detected in the oral fluid sample and this pen was the only pen in the nurseries in which *A. pleuropneumoniae* was found. However, *A. pleuropneumoniae* was widely distributed in the finishers. Porcine cytomegalovirus was detected in many of the pens, but with the lowest Cq values in the nurseries. Porcine circovirus type 2 and *S. suis* type 2 were detected in all pens except for one, and PCV2 was present with Cq values below 16 in several of the pens with the majority in the finishers. Swine influenza A virus was detected in all age groups in the nurseries and was also detected in the beginning of the finisher period. One of the samples collected in the nurseries in September, was positive for A(H1)pdm09. Porcine circovirus type 3 was present in all age groups. Diarrhoea seemed to be a problem in this herd, since 28 diarrhoeic events (Additional file 1I and 1J) distributed between the nurseries and finishers were observed. *Brachyspira pilosicoli* and *L. intracellularis* were mainly detected in the mid to late nursery period in the faecal sock samples, while *B. pilosicoli* was found in every pen in the finishers and here *L. intracellularis* was only found in the beginning of the period. *Escherichia coli* F18 was detected in the nursery pigs 15–17 days after weaning in the 3 months, while *E. coli* F4 was only present in November.

### Pathogen findings and clinical signs

This section describes the presence and dynamics of pathogens across the production units and, when relevant, the association between pathogens and coughing or diarrhoeic events.

#### Swine influenza a virus

**Fig. 1:**
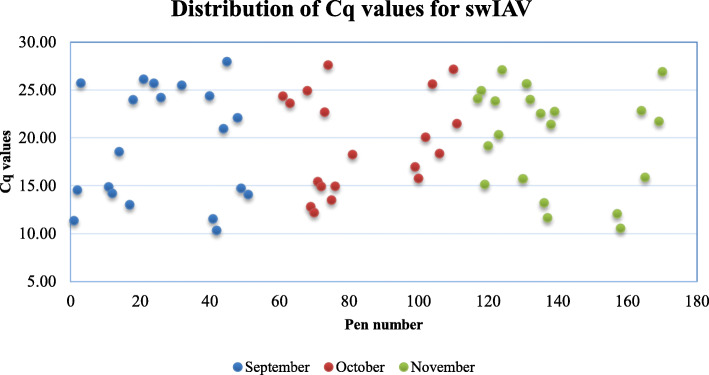
Quantification cycle values for the swIAV positive samples from the three sampling months (negative pens are not shown)

Swine influenza A virus was found in 60 of the 172 pens (34.9%) and it was detected in all ten production units. The infection patterns varied between the units and age groups. Swine influenza A virus was mostly present shortly after weaning, and only sporadically later in the nursery period. In the finishers, only a few pens were found positive and mainly in the beginning of the period. Six out of eight pens, in which coughing events were registered, were found to be positive for swIAV and an association between coughing events and swIAV detection were found (*p* = 0.02). Furthermore, to investigate whether the level of swIAV was associated to the coughing events different cut-off Cq values were tested (Table 2). No association between coughing events and the level of swIAV was found with cut-off values of Cq 16 (*p* = 0.08) and 18 (*p* = 0.10), while with a cut-off value of Cq 20 an association was observed (*p* = 0.03). The Cq values for all the positive swIAV findings are plotted in Fig. 1.

**Table 2:** *p*-values (Fishers exact probability test) calculated for different cut-off Cq values for respiratory viruses

Coughing event	+/−	Cq ≤ 16	Cq ≤ 18	Cq ≤ 20	Cq ≤ 22
*p*-value (swIAV)	0.02	0.08	0.10	0.03	na
*p*-value (PCMV)	1.00	0.0001	0.002	0.02	0.21

*na* no analysis

### Porcine cytomegalovirus

**Fig. 2:**
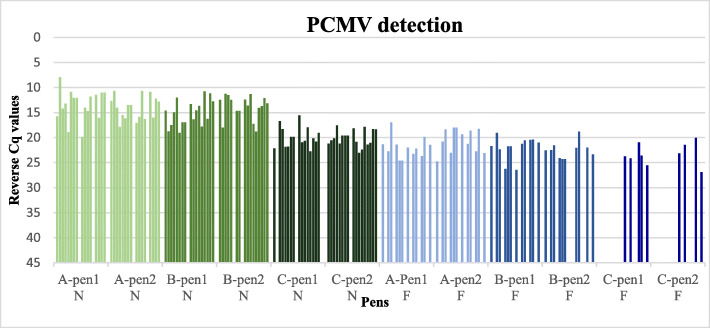
Reverse quantification cycle values for the positive PCMV samples from all the sampled pens. The samples are listed from youngest to oldest. A, B and C refer to the youngest, mid and oldest pigs, respectively, in both nurseries (N) and finishers (F). The y-axis shows the reverse Cq values

**Fig. 3:**
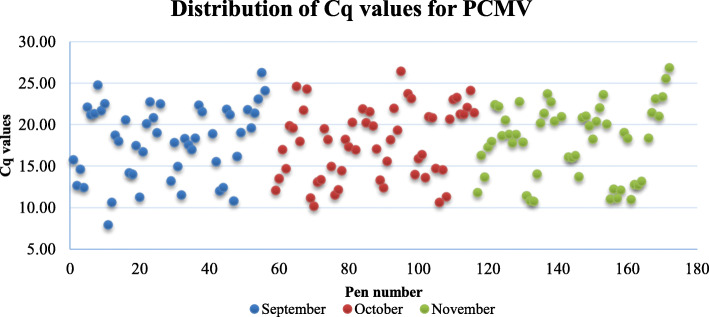
Quantification cycle values for the PCMV positive samples from each of the three sampling months (negative pens are not shown)

Porcine cytomegalovirus was detected in all ten production units and it was detected in 159 of the 172 pens (92.4%). In general, the detection of PCMV was highly consistent with the lowest Cq values in the early to mid nursery period and with higher Cq values in the samples collected from six to 8 weeks after weaning and until slaughtering (Fig. 2). Porcine cytomegalovirus was detected in the eight pens in which coughing events were registered with Cq values between 8.0 and 16.3. Furthermore, there was an association between the level of PCMV and coughing events with cut-off values of Cq 16 (*p* = 0.0001), 18 (*p* = 0.002) and 20 (*p* = 0.02), while no association was observed with a cut-off value of 22 (*p* = 0.21) (Table 2). The Cq values for all the positive findings of PCMV are plotted in Fig. 3.

In general, PCMV was present in all the pens where swIAV was detected expect for one pen (4F). Furthermore, PCMV with Cq values ≤20 was also detected in the six “coughing event” pens, which were positive for swIAV, and an association between these two viruses and coughing events was found (*p* = 0.004).

#### Porcine circovirus type 2

Porcine circovirus type 2 was detected in all ten production units, but with different patterns. For the oral fluid samples, the virus was detected in 103 of the 172 pens (59.9%), while for the faecal sock samples it was detected in 70 of the 174 pens (40.2%). Porcine circovirus type 2 was detected in four of the “coughing event” pens with Cq values above 23, and no association between detection of PCV2 and coughing event was observed (*p* = 0.72). Since it has been shown that PCV2 can be related to intestinal disease, its association with diarrhoeic events was also investigated [24]. Porcine circovirus type 2 was detected in 41.6% of the pens where diarrhoeic events were observed, with Cq values between 10.2 and 27.5. No association was observed between the presence of PCV2 and diarrhoeic events (*p* = 0.87), even when using different cut-off values (Table 3).

**Table 3:** *p*-values (chi-square test) calculated for different cut-off Cq values for the intestinal pathogens

Diarrhoeic event	+/−	Cq ≤ 16	Cq ≤ 18	Cq ≤ 20	Cq ≤ 22	Cq ≤ 24	Cq ≤ 26	Cq ≤ 27
p-value (PCV2)	0.87	0.30	0.48	0.08	0.14	na	na	na
p-value (*B. pilosicoli*)	0.06	na	0.005	0.001	0.007	0.004	0.03	0.06
p-value (*L. intracellularis*)	0.12	na	0.006	0.008	0.03	0.01	0.91	na
p-value (*E. coli* F4)	0.43	na	na	0.82	0.43	na	na	na
p-value (*E. coli* F18)	0.06	na	na	na	0.82	0.58	na	na
p-value (Rotavirus A)	0.0002	na	na	0.3	0.009	0.007	na	na

*na* no analysis

### Porcine circovirus type 3

Porcine circovirus type 3 was detected in all production units with the majority of Cq values above 20. The virus was detected sporadically in most of the units, but more frequently in herd 3 (unit 3 N and 3F). In the oral fluid samples, PCV3 was detected in 93 of the 172 pens (54.1%), while for the faecal sock samples, the virus was found in 28 of the 174 pens (16.1%). Porcine circovirus type 3 was detected in five of the “coughing event” pens, where four of the pens had Cq values above 25, while the last one had a Cq value of 19, and no association was observed between the presence of PCV3 and coughing events (*p* = 0.73). Porcine circovirus type 3 was present in 16.9% of the pens, where diarrhoeic events were registered, with Cq values above 23.

#### Actinobacillus pleuropneumoniae

*Actinobacillus pleuropneumoniae* was detected in five of the ten production units and in 30 of the 172 pens (17.4%). *Actinobacillus pleuropneumoniae* was found in three of the eight pens, where coughing events were observed, with Cq values above 24. There was no association between *A. pleuropneumoniae* positive pens and coughing events (*p* = 0.15).

#### Mycoplasma hyohrinis

**Fig. 4:**
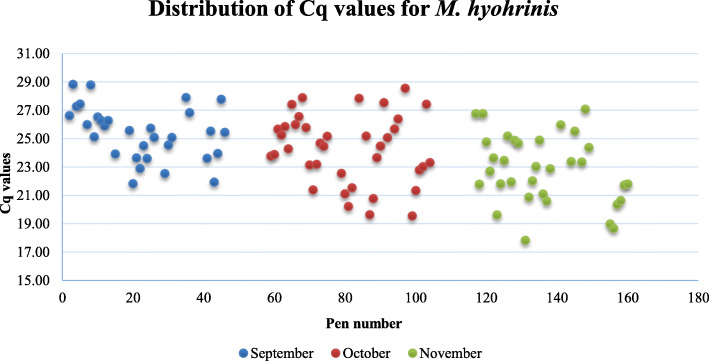
Quantification cycle values for the *M. hyohrinis* positive samples from each of the three sampling months (negative pens are not shown)

*Mycoplasma hyohrinis* was detected in eight out of the ten production units and in 102 of the 172 pens (59.3%). In production unit 1 N, 1F, 2 N, and 5 N almost all pens were positive, while a more sporadic distribution was found in production unit 3 N, 3F, 4 N, and 4F. *Mycoplasma hyohrinis* was not detected at all in unit 6 N and 6F. *Mycoplasma hyorhinis* was present in seven of the “coughing event” pens with Cq values above 20 and no association between detection of *M. hyorhinis* and coughing event was found (*p* = 0.08). In Fig. 4, Cq values from the positive samples were plotted and a decrease in Cq values was observed during the 3 months, with the highest mean Cq in September (25) and the lowest mean Cq in November (23). An one-way ANOVA test confirmed that the mean Cq values for the 3 months were not equal. Furthermore, a t-test analysis showed that there was a significant difference between the mean Cq values for the 3 months: September and October (*p* = 0.03), October and November (*p* = 0.01) and September and November (*p* = 0.00001).

#### Streptococcus suis type 2

*Streptococcus suis* type 2 was detected in all ten production units and in 170 of the 172 pens (98.8%). *Streptococcus suis* type 2 was found in all eight coughing event pens with Cq values above 21. No association was found between *S. suis* type 2 detection and coughing event (*p* = 0.91).

#### Mycoplasma hyopneumoniae, Bordatella bronchiseptica and *Pasteurella multocida*

*Mycoplasma hyopneumoniae* was only found in two pens (1.2%) and both in production unit 6F in the sampling from October. *Bordetella bronchiseptica* and *P. multocida* were detected in 43 (25.0%) and 26 (15.1%) of the 172 pens, respectively. However, none of these bacteria were detected in pens with coughing events.

#### Brachyspira pilosicoli

*Brachyspira pilosicoli* was found in 69 of the 174 pens (39.7%) and was generally found from mid nursery to late finishers period. *Brachyspira pilosicoli* was detected in all production units except for 1 N and 1F, and it was present in 37 (48.1%) of the tested pens, in which a diarrhoeic event was observed. An association between diarrhoeic event and the presence of *B. pilosicoli* was observed when using a cut-off Cq value ≤26 (*p* = 0.03) (Table 3).

#### Lawsonia intracellularis

*Lawsonia intracellularis* was detected in 71 of the 174 pens (40.8%) and was found mainly in the mid to late nursery and finisher period. *Lawsonia intracellularis* was detected in 37 (48.1%) of the pens, where a diarrhoeic event was observed. For *L. intracellularis*, an association between the presence of the bacteria and diarrhoeic events was found when using a cut-off Cq value ≤24 (*p* = 0.01) (Table 3).

#### *Escherichia coli* F4 and F18

*Escherichia coli* F4 and *E. coli* F18 were detected in 33 (19.0%) and 50 (28.7%) of the 174 pens, respectively, and mainly in the beginning of the nursery period, although a sporadic detection was also observed in the finishers. In general, *E. coli* F18 was detected more frequently than *E. coli* F4 in the production units except for 2 N and 4 N. *Escherichia coli* F4 was present in 12 (15.6%) of the tested pens, where a diarrhoeic event was observed, while for *E. coli* F18 the number of pens was 16 (20.8%). For both *E. coli* F4 and F18, no association was found to pens with diarrhoeic events (*p* = 0.43 and *p* = 0.06, respectively), even when using different cut-off Cq values (Table 3).

### Rotavirus a

Rotavirus A was detected in all production units and was found in 94 of the 174 pens (54.0%). In general, rotavirus A was most frequently detected in the beginning of the nursery period, in which also the lowest Cq values were found. In the youngest age group of the nurseries, only one pen (4 N) was negative. In the finishers, rotavirus A was only detected sporadically. The virus was detected in 29 (37.7%) of the pens, where a diarrhoeic event was observed. No association between the presence of rotavirus A and diarrhoeic events was found when using a cut-off Cq value ≤20 (*p* = 0.3), however, an association was observed when including all positive Cq values (*p* = 0.0002) (Table 3).

## Discussion

In the present study, three monthly samplings were conducted in six nursery and four finisher production units to investigate the value of continuous screening for selected respiratory and enteric viral and bacterial pathogens in different age groups. Oral fluid and faecal sock samples were collected and analysed using the high-throughput diagnostic system described elsewhere [22]. The use of a high-throughput rtPCR platform, in which multiple samples can be analysed in different assays simultaneously, provides new possibilities for conducting extended pathogen detection at a limited cost. The aim of the present study was to investigate the pathogen patterns in ten production units over time and compare the findings with the observed clinical signs of coughing and diarrhoea.

In herd 1 (1 N and 1F), swIAV was circulating in all age groups, which was probably due to the continuous flow of pigs in the nursery rooms and/or the continuous production of finishers without washing and disinfection of pens between batches. Only new gilts and gilts prior to farrowing were vaccinated against swIAV (Respiporc FLU3, IDT), however, vaccination of piglets could be considered based on the screening results. Furthermore, *E. coli* F4 and *E. coli* F18 were often detected in this herd without correlation to disease. However, enteritis can be present in a herd without causing clinical signs [25], and therefore, vaccination targeting *E. coli* for nursery pigs may be beneficial.

In herd 2 (2 N), swIAV was present in all age groups with low Cq values but the A(H1)pdm09 subtype was not detected at any sampling point. The herd vaccinated against A(H1 N1)pdm09 (RESPIPORC FLUpan H1 N1, CEVA), but this vaccine does not cross protect against enzootic Danish swIAVs, and therefore, vaccination against other swIAV subtype(s) could be considered. In this herd, newly weaned pigs were fed liquid feed and extra water in troughs to optimize weaning, which may contribute to the low levels of intestinal pathogens and clinical diarrhoea observed in general.

In herd 3 (3 N and 3F), there was a long-term history of swIAV infection and, therefore, an intensive vaccination protocol was carried out, in which piglets, new gilts, gilts and sows were vaccinated (Respiporc FLU3, IDT). Despite the vaccination, swIAV was still present in the early to mid nursery period and in the finishers 14–16 days after arrival. The continued circulation of swIAV could be due to infection with a heterologous subtype(s) not included in the vaccine [26] or that the effect of the piglet vaccination was short lasting [27]. Therefore, more detailed genetic characterisation of the circulating swIAV subtype(s) should be carried out to determine the level of genetic identity between the circulating field strain and the vaccine strain. Furthermore, it could be considered to vaccinate sows 3–4 times a year to ensure high levels of immunity in the sow herd.

In herd 4 (4 N and 4F), the enteric pathogens *E. coli* F4, *E. coli* F18, *B. pilosicoli* and *L. intracellularis* were detected in several of the pens. In addition, PCV2 was present in all age groups, and especially in the finishers. Furthermore, the average daily weight gain in the finishers was below the Danish national average. Thus, these data implies that PCV2 has a negative impact in this herd, and therefore, vaccination against PCV2 could be initiated.

In herd 5 (5 N), a comprehensive vaccination protocol was applied against swIAV (Respiporc FLU3, IDT), including vaccination of piglets, new gilts before introduction, gilts and sows. Despite this vaccination regime, swIAV was still detected, which could indicate that the effect of vaccination was not optimal [26, 27]. To address this, more detailed genetic characterisation of the circulating swIAV strains should be performed to secure that the vaccine elicited cross protection. Furthermore, the herd had a poor productivity (ADWG at 385 g/day) and a nursery mortality rate of 4% was found based on the data from the productivity report (Table 1). Porcine circovirus type 2 vaccination was performed at weaning, but still moderate levels of PCV2 were found in the youngest nursery pigs and therefore it cannot be excluded that PCV2 has an influence on the poor productivity. Thus, it may be beneficial in this herd to perform PCV2 vaccination at an earlier time point.

In herd 6 (6 N and 6F), new gilts were vaccinated against A(H1 N1)pdm09 (RESPIPORC FLUpan H1 N1, CEVA) and swIAV (Respiporc FLU3, IDT) before introduction. A(H1)pdm09 was detected in one pen, while other swIAV subtype(s) was found to be present sporadically in several of the pens, mainly in the nursery herd. This may be because the level of virus were too low to be detected by the A(H1)pdm09 assay. Therefore, more detailed genetic characterisation of the circulating swIAV subtype(s) should be performed on new samples (nasal swabs) to substantiate the choice of vaccine. Porcine circovirus type 2 was widely distributed in both the nurseries and finishers and in high levels which could lead to systemic disease and lead to decreased productivity [19]. Vaccination against PCV2 was not applied in this herd, but based on the findings in the present study, vaccination before weaning could be considered to control PCV2.

*Mycoplasma hyopneumoniae* was detected in two pens in the finisher unit despite vaccination. In the finishers, a mortality rate of 4.5% was found based on the data from the productivity report. The mortality could be due to concurrent circulation of several pathogens, but more investigations are needed to confirm this. A high stocking density was often noticed during the samplings, which could contribute to the on-going circulation of these pathogens. High occurrence of diarrhoea seemed to be a clinical issue in both nurseries and finishers with a clear pattern. *Escherichia coli* F4 and/or *E. coli* F18 were found in the beginning of the nursery period followed by *L. intracellularis* and *B. pilosicoli,* which persisted until slaughter. Common practice in this herd was to use water medication pen wise, however, an extension to batch medication may be more effective. Furthermore, focus should be on optimizing diets, hygiene, stocking density in the finishers. *Escherichia coli* F4 and *E coli* F18 vaccination could be considered.

The coughing events observed in the present study seemed to correlate with the presence of swIAV (*p* = 0.02) or PCMV (Cq ≤ 20) (*p* = 0.02) or a combination of these (*p* = 0.004). The association between coughing and isolation of swIAV has been shown in other studies [26, 28, 29]. However, the clinical effects of PCMV are not clear and a cut-off Cq value was also needed in the present study in order to obtain an association to coughing events. To our knowledge, co-infection with swIAV and PCMV has not previously been described, and further studies are needed to examine the interaction between the two viruses.

Swine influenza A virus was detected in all the production units, despite that all herds, except for herd 4, vaccinated against swIAV using different vaccination strategies. Among the units, different infection patterns were observed, however, the most typical pattern was high levels of swIAV just after weaning, which could indicate short-term duration of maternal immunity or antigen mix-match between the vaccine and herd strain [30, 31].

Porcine circovirus type 2 was found with a high prevalence in the production units 4F, 5 N, 6 N, and 6F. Although no correlation to neither coughing nor diarrhoeic events was evident, PCV2 might still act sub-clinically. Furthermore, reasonable good management practice was seen in all units, which combined with high health status, could minimize the impact of PCV2. The role of PCV2 in PRDC is still up for discussion. Studies suggest that PCV2 lung lesions do not exist without systemic infection and may only participate in PRDC sub-clinically [32, 33]. This could explain the missing correlation between PCV2 and clinical respiratory signs in the present study. Furthermore, a study suggested that PCV2 is more often found in connection with PRRSV than with swIAV and *M. hyopneumoniae* [8], however, PRRSV was not detected in this study.

Porcine circovirus type 3 is a novel discovered virus, which was described for the first time in 2016 in USA [34]. Porcine circovirus type 3 has been shown to be widely distributed in Europe but also worldwide [35, 36] and the virus was also found in all age groups in both nurseries and finishers in the current study. Porcine circovirus type 3 was primarily detected with relatively high Cq values and it did not seem to correlate to events of respiratory disease.

There is sparse documentation on the predictive value of detecting bacteria in oral fluid samples and therefore oral fluid is recommended only as a screening tool and the detection of these bacteria should be confirmed by traditional diagnostic tests (i.e. culturing from lungs of dead pigs or PCR on lung tissue). *Mycoplasma hyorhinis, S. suis* and *P. multocida* are all considered to be secondary invaders in relation to PRDC and commensals present in both healthy and diseased pigs [1, 9]. In this study, *S. suis* type 2 and *M. hyorhinis* were indeed detected with relatively high Cq values in all pens (except 6 N and 6F for *M. hyorhinis*) and no clinical signs could be correlated to the detection of these two pathogens. *Pasteurella multocida* was found more sporadically. The bacterium was also detected with high Cq values and it was not found in relation to clinical disease. Interestingly, the mean Cq values of *M. hyorhinis* differed significantly between all three sampling months, with the highest Cq mean recorded in September and the lowest Cq mean in November. This could indicate a seasonal variation in the infection pressure of *M. hyorhinis* although the clinical effect is unknown. A benefit of continuous health monitoring could potentially be the detection of such seasonal variances for more of the analysed pathogens if evaluated for an extended period.

*Actinobacillus pleuropneumoniae*, *M. hyopneumoniae* and *B. bronchiseptica* act as primary pathogens, providing optimal conditions for secondary pathogens [1, 9]. *Actinobacillus pleuropneumoniae* was only found in the oral fluids in production units already declared positive and was only detected with high Cq values, which could indicate that they were only carrier animals, harbouring *A. pleuropneumoniae* in the tonsils. Another explanation could be that the sensitivity of detecting *A. pleuropneumoniae* by PCR in oral fluid compared to lungs is considered low [37]. However, the bacterium was not detected in correlation to clinical signs in this study. *Mycoplasma hyopneumoniae* was rarely detected in this study. A study found limited sensitivity of detecting *M. hyopneumoniae* in oral fluid, which could lead to an underestimation in positive pens [3]. *Bordetella bronchiseptica* was found sporadically with high Cq values and it was not found in relation to clinical disease.

For some of the production units, a clear pattern of enteric pathogens was observed, in which *E. coli* F4 and/or *E. coli* F18 were present in the early nursery period, *L. intracellularis* in the mid nursery to early finishing period and *B. pilosicoli* in the late nursery to late finisher period. This distribution of these bacteria is comparable to others findings [14, 38, 39]. For other of the production units, no specific pattern was found. Diarrhoeic events observed in the present study seem to be associated with different bacteria in different age groups and with a similar pattern. However, no association between diarrhoeic events and the presence of *E. coli* F4 and *E. coli* F18 was found. On the other hand, an association was observed for *L. intracellularis* (Cq ≤ 24) and *B. pilosicoli* (Cq ≤ 26). Furthermore, rotavirus A was detected in all herds and mainly in the nurseries. This virus is known to be endemic in pig herds and has been associated with acute gastroenteritis in young animals [16]. In the present study, rotavirus A was found in several of the pens affected by diarrhoeic events, however, an association between diarrhoeic event and the presence of the virus was only observed when including all Cq values (*p* = 0.0002). Since rotavirus A only seems to pose a problem when including the high Cq values, this virus was probably not the cause of diarrhoea in the affected pens.

Diarrhoeic events were also observed in pens where no intestinal pathogens were found indicating that the course was non-infectious or caused by pathogens, which were not included in the high-throughput rtPCR analysis. Another study found that approximately 50% of the investigated pigs suffering from diarrhoea were negative for pathogenic intestinal bacteria when using rtPCR [40].

In general, the findings in the present study were based on Cq values, which gives an indication of the initial copy numbers of the target. However, quantitative measurements of pathogen load, including e.g. copies/g faeces, will provide a more informative result [41]. For several of the intestinal bacteria, cut-off values have been proposed [13], and these values can be used to examine if a given bacteria is the main reason for the observed clinical disease or not. It should, though, be kept in mind that the interpretation is only valid when using the assays validating the associations. Copy numbers for a given pathogen may differ when using different assays (e.g. Hjulsager et al., 2009 [42]) and even when using the same assays in different laboratories.

The results presented here represent sampling of herds during the autumn which biased the findings because some pathogens have a seasonal distribution (eg. *M. hyopneumoniae* is more prevalent during the winter months [43, 44]) which should be taken into consideration when the results are interpreted and compared.

## Conclusions

The vision of the diagnostic system tested in the present study, was that the herd veterinarian includes the results of the diagnostic screenings as a tool in the herd health management by benchmarking the monthly data on the presence and dynamics of pathogens with figures on productivity, feed consumption/feed plans, clinical symptoms and antibiotic consumption. By that the herd veterinarian will be able to identify the underlining course of impaired production figures or poor health. Furthermore, in case of acute outbreak of clinical disease or sudden change in performance, samples are commonly submitted for diagnostic examinations, however, the results of these tests can be difficult to interpret because most of the potential pathogens are often circulating in the herds with limited clinical impact. If screening of the herd had been performed during the months prior to the acute outbreak, the results of the diagnostic investigation performed in relation to the outbreak situation could be compared to these herd-specific historical data and by that identify if a given pathogen has been introduced or has changed dynamics. In all of the ten production units included in the present study, the screening data identified unexpected pathogen patterns and by that identified potential targets for preventive measures to increase the health and/or productivity.

The use of the high-throughput method in combination with information about clinical signs, productivity, health status and antibiotic consumption, presents a new and innovative way of diagnosing and monitoring pig herds and even to a lower cost than the traditional method. Recently, this method have been commercialized by the Centre for Diagnostics at the Technical University of Denmark (www.diagnostik.dtu.dk) and thereby, provide a new innovative tool for implementation and adjustment of preventive measures with the goal to reduce the use of antibiotics in pig herds.

## Materials and methods

### Study design and herd inclusion criteria

The study was carried out as a repeated cross-sectional study in six herds, which covered ten pig production units located on Zealand, Denmark. Six nursery (1 N, 2 N, 3 N, 4 N, 5 N and 6 N) and four finisher (1F, 2F, 3F and 6F) units from six different herd systems were included in the study. Herds 2, 3, 4, 5 and 6 were included in the Danish SPF-system [23], while the SPF status of herd 1 was unknown. The target populations of each production unit consisted of three different age groups in the nursery and finisher pigs. For each production unit, sampling and clinical registrations were conducted monthly over a 3 months period from September to November. General information on the production units and their vaccination strategies was obtained from questionnaires completed by the herd owners (Table 1).

### Sampling strategy and clinical observations

**Fig. 5:**
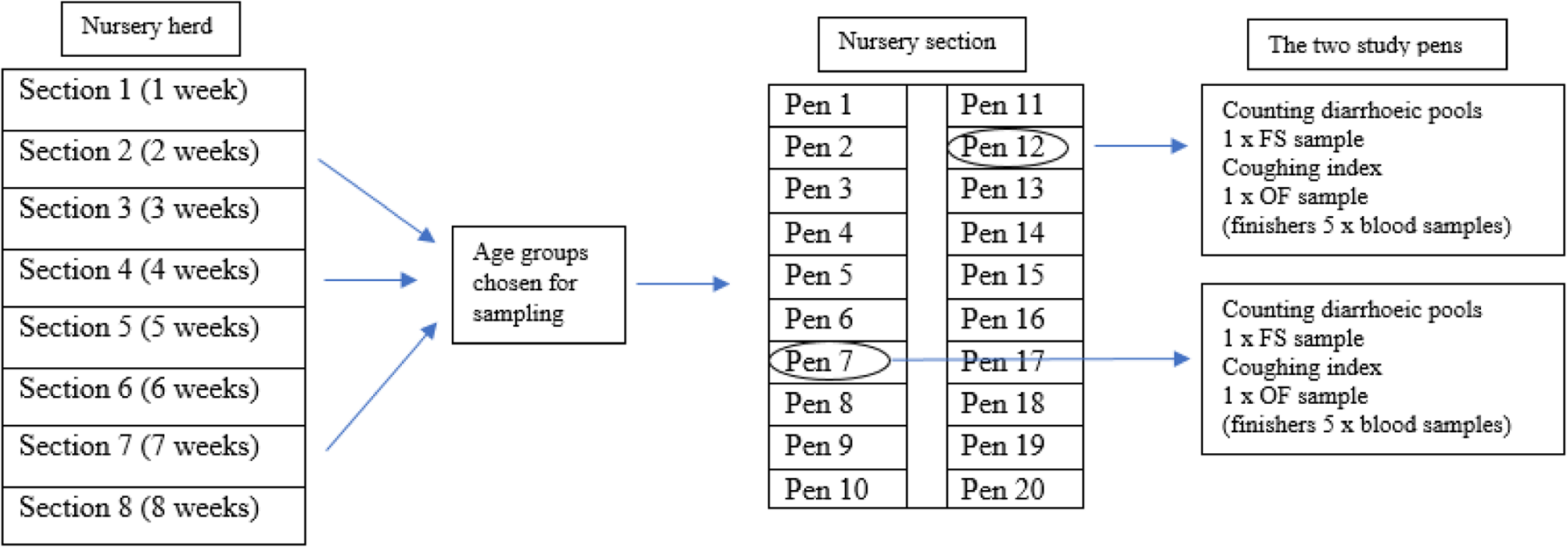
Schematic illustration of how age groups and pens were selected. Here, a nursery herd is used as an example. FS: faecal sock samples, OF: oral fluid samples

At each sampling, three different age groups in each production unit were sampled (Fig. 5). In the nursery units, pigs were sampled approximately two, five, and 7 weeks after weaning. In the finisher units, except for unit 1F, pigs were sampled approximately two, six, and 10 weeks after achieving a weight of 30 kg. In the finisher unit for herd 1 (1F), only two age groups were sampled due to continuous flow and no clear separation of age groups. Samplings were always conducted at the same location/unit if a farm consisted of multiple finisher units, occasionally resulting in a deviation from the sampling age with up to 2 weeks. To reduce the spread of disease, the youngest pigs were always sampled first. Two pens from each age group were randomly included in the study, representing that age group. By random sampling, one pen was first chosen. To minimize environmental factors and to maximize the distance between the study pens, the other pen was chosen by turning to the pen just opposite of the aisle and then move a defined number of pens downwards, calculated by dividing the number of pens in a row by two (Fig. 5). In unit 5 N, four nursery pens were included per age group due to the small numbers of pigs housed per pen. Before nucleic acid extraction, the samples from the four pens were pooled two by two within each age group – giving two pens per age group. Hospital pens were excluded from the study. Due to the cross-sectional study design, the same pens were not targeted to be sampled at the next sampling month, although this could happen by coincidence.

At each sampling, the coughing index, which is a method to quantify coughing in groups of pigs, was calculated [45]. The pigs in the study pens were counted and forced to move if sleeping. Coughs were then counted for 3 min. If the same pig was coughing more than once within a 10 s period, this coughing bout was noted but only counted once in the total number of coughs. The coughing index was calculated as: Coughing index = total number of coughing bouts/number of pigs*total time of observation (min) [45]. Based on the coughing index, a coughing event was defined to determine if the pen was a coughing or a non-coughing pen. If the coughing index was ≥0.2, the pen was defined as having a coughing event [28]. Similarly, the incidence of diarrhoea was evaluated at each sampling by counting the number of diarrhoea blobs in the study pens. Assessment of the faecal consistency was done by using a descriptive classification scale described by Pedersen and Toft, 2011 [46]. A diarrhoeic index was calculated based on the number of diarrhoea blobs in a pen and if the diarrhoeic index was ≥0.1, the pen was defined as having a diarrhoeic event [47].

### Sampling of oral fluid and faecal sock samples

Oral fluid samples were collected from nursery and finisher pigs through the application of an oral fluid sampling kit (Dianova, Kgs. Lyngby, Denmark). Oral fluid sampling was performed pen-wise by tying a cotton rope to the gate of the pen 20–30 cm above the floor (height adjusted to the size of the pigs). For approximately 30 min, the pigs were allowed to chew on the rope and thereby deposit oral fluid in the rope. After 30 min, the rope was collected and placed in a plastic bag, squeezed and oral fluid extracted in a 10 mL centrifuge tube. To minimize cross-contamination between samples, disposable gloves and new scalpels (used to cut the plastic bags) were used for each pen.

In addition, faecal sock samples were collected from nursery and finisher pigs by means of a sock sampling kit (Dianova, Kgs. Lyngby, Denmark). One sock sampling kit was used per pen. Faecal sock samples were acquired by treading through the faecal contaminated part of the pen wearing the sampling socks as previously described [48].

Oral fluid and faecal sock samples were marked with specific identification numbers with information on pen, herd, age group, sample type, and date. All samples were stored in a polystyrene box containing freezer packs before delivery to the Centre for Diagnostics, Technical University of Denmark. After delivery, all samples were kept under cooled conditions in a refrigerator at approximately 5 degrees for a maximum of 48 h until preparation. Oral fluid samples were dispensed and stored at − 80 °C for RNA and DNA extraction. For each of the faecal sock samples, a 10% faeces dilution was prepared in phosphate buffered saline and stored at − 80 °C for RNA and DNA extraction.

### Nucleic acid extraction

Oral fluid samples were extracted using the extraction robot QIAcube HT (QIAGEN, Hilden, Germany) and the Cador pathogen 96 QIAcube HT kit (QIAGEN). The Cador pathogen 96 QIAcube HT protocol (QIAGEN) was applied with the following modifications; the input volume was increased from 200 to 400 μL and the volume of lysis buffer VXL was increased from 100 to 200 μL. Before extraction, 1000 μL of each oral fluid samples were centrifuged for 5 min at 9000 x g at room temperature (15–25 °C) and 400 μL of the supernatant was used for extraction together with positive (cell culture lysates (viruses) and bacterial cultures) and negative (nuclease-free water, Amresco) controls run in parallel. The nucleic acid extractions were stored at − 80 °C until further analysis.

The 10% faeces dilution samples were extracted using the QIAsymphony SP system (QIAGEN) extraction robot and the QIAsymphony DSP virus/pathogen mini kit (QIAGEN) following the manufacturer’s instructions. For this, the protocol Complex200_V5_DSP was used with an elution volume of 110 Μl*. prior* to the nucleic acid extraction, one 5 mm steel bead was added to each sample following which the samples were homogenized in a Tissuelyser II (QIAGEN) for 20 s at 15 Hz. The homogenate was then centrifuged for 90 s at 6700 x g and 350 μL of the supernatant was used for nucleic acid extraction together with positive (cell culture lysates (viruses) and bacterial cultures) and negative (nuclease-free water, Amresco) controls run in parallel. The nucleic acid extractions were stored at − 80 °C until further analysis.

### Pathogen detection by high-throughput real-time PCR

Extracted oral fluid and faecal sock samples were both reverse transcribed/pre-amplified and pre-amplified as previously described [22]. For high-throughput rtPCR amplification, the BioMark 48.48 dynamic array (DA) Integrated Fluidic Circuit system (Fluidigm, South San Francisco, USA) was used, which combines 48 pre-amplified samples with 48 assays for 2304 individual and simultaneous rtPCR reactions. The rtPCR assays and procedure used for the high-throughput rtPCR analysis in the present study have previously been described and validated in the study by Goecke et al., 2020 [22]. The rtPCR assays were performed in duplicates for each sample. Three positive PCR controls, containing mixtures of positive RNA/DNA for the included assays, two non-template controls (nuclease-free water, Amresco) and a non-template cDNA/pre-amplification and a pre-amplification control (nuclease-free water, Amresco) were included on each 48.48 DA to control for non-specific amplification and contamination.

For the swIAV analysis, two different assays were included in the high-throughput rtPCR analysis, one general swIAV assay detecting all known subtypes of swIAV and an assay specific for the HA gene of human pandemic H1 N1 strain (A(H1 N1)pdm09). Thus, a positive result for swIAV are reported as either “swIAV” if the general swIAV assay was positive and “A(H1)pdm09” if both assays were positive for a given sample.

### Statistical analysis

The association between the presence of a pathogen and clinical signs in a pen was investigated using chi-square test or Fishers exact probability test, if cell frequencies were equal to or greater than five. For the respiratory pathogens, the Fishers exact probability test (two-tailed) was used with the following parameters; presence vs. absence of pathogens and coughing event vs. no coughing event. For the intestinal pathogens, the chi-square test (with Yates correction) was used with the following parameters; presence vs. absence of pathogens and diarrhoeic event vs. no diarrhoeic event. To investigate whether the pathogen level was associated with coughing or diarrhoeic events different cut-off Cq values were tested in the chi-square test and Fishers exact probability tests. For comparison of the mean Cq values between September, October and November the one-way analysis of variance (ANOVA) test and the t-test were used. The analyses were performed using Graph Pad Prism version 7.0.

## Supplementary information

**Additional file 1.** High-throughput rtPCR results for the ten production units. The table shows the quantification cycle values from the high-throughput rtPCR analysis for the oral fluid (OF) and faecal sock (FS) samples, which were collected monthly over a 3 months period from September to November from ten production units (Table 1A-J). Pens where a coughing event was recorded are marked with bold and italics, while pens where a diarrhoeic event was observed are marked with bold and underscore. An empty box in the table indicates a negative rtPCR results.

## Data Availability

The datasets used and/or analysed during the current study are available from the corresponding author on reasonable request. The dataset supporting the conclusions of this article is included within the article (and its additional file).
