# Supplementary material for: Objective pathogen monitoring in nursery and finisher pigs by monthly laboratory diagnostic testing
Source: Porcine Health Manag. 2020 Sep 8;6:23. doi: 10.1186/s40813-020-00161-3 (PMC7476771; doi:10.1186/s40813-020-00161-3)
Supplement: Supplementary file 1 — Additional file 1. High-throughput rtPCR results for the ten production units. The table shows the quantification cycle values from the high-throughput rtPCR analysis for the oral fluid (OF) and faecal sock (FS) samples, which were collected monthly over a 3 months period from September to November from ten production units (Table 1A-J). Pens where a coughing event was recorded are marked with bold and italics, while pens where a diarrhoeic event was observed are marked with bold and underscore. An empty box in the table indicates a negative rtPCR results. [file 40813_2020_161_MOESM1_ESM.docx]

Additional file 1

| **A) 1N – oral fluid (OF) and faecal sock (FS) samples** | | | | | | | | | | | | | | | | | | | |
| --- | --- | --- | --- | --- | --- | --- | --- | --- | --- | --- | --- | --- | --- | --- | --- | --- | --- | --- | --- |
| Sampling date | | September | | | | | | October | | | | | | November | | | | | |
| Days after weaning | | 10 | | 25 | | 45 | | 19 | | 26 | | 47 | | 10 | | 24 | | 45 | |
| Pathogen | Material | Pen 1 | Pen 2 | **Pen 1** | Pen 2 | **Pen 1** | **Pen 2** | Pen 1 | **Pen 2** | Pen 1 | Pen 2 | Pen 1 | **Pen 2** | **Pen 1** | ***Pen 2*** | **Pen 1** | Pen 2 | **Pen 1** | **Pen 2** |
| swIAV | OF | 11.4 | 14.5 | 25.7 |  |  |  |  |  | 24.4 |  | 23.6 |  | 24.1 | 24.9 | 15.1 | 19.2 |  | 23.9 |
| A(H1)pdm09 | OF |  |  |  |  |  |  |  |  |  |  |  |  |  |  |  |  |  |  |
| PRRSV-1 | OF |  |  |  |  |  |  |  |  |  |  |  |  |  |  |  |  |  |  |
| PRRSV-2 | OF |  |  |  |  |  |  |  |  |  |  |  |  |  |  |  |  |  |  |
| PCMV | OF | 15.8 | 12.7 | 14.6 | 12.5 | 22.1 | 21.2 | 12.1 | 13.5 | 17.0 | 14.7 | 19.9 | 19.6 | 11.8 | 16.3 | 13.7 | 17.3 | 18.0 | 22.4 |
| *A. pleuropneumoniae* | OF |  |  |  |  | 24.4 |  |  |  |  |  |  |  |  | 26.8 |  |  |  |  |
| PCV2 | OF |  |  |  |  |  |  | 27.5 |  | 21.6 |  |  |  |  |  | 27.0 |  | 23.3 |  |
| *S. suis* type 2 | OF | 22.5 | 22.8 | 26.2 | 26.6 | 21.9 | 22.2 | 24.4 | 22.6 | 22.9 | 23.6 | 24.3 | 23.22 | 24.6 | 21.1 | 23.1 | 24.4 | 17.4 | 19.6 |
| *B. bronchiseptica* | OF |  |  |  |  | 25.9 |  |  |  | 27.7 |  | 25.5 |  |  |  |  |  |  |  |
| PCV3 | OF |  |  | 26.6 |  |  |  | 25.1 | 24.8 |  |  |  |  | 17.3 | 18.7 | 27.9 |  |  | 30.0 |
| PPV | OF |  |  |  |  |  |  |  |  |  |  |  |  |  |  |  |  |  |  |
| *M. hyopneumoniae* | OF |  |  |  |  |  |  |  |  |  |  |  |  |  |  |  |  |  |  |
| *M. hyorhinis* | OF |  | 26.6 | 28.8 | 27.3 | 27.5 |  | 23.8 | 23.9 | 25.7 | 25.3 | 25.9 | 24.3 | 26.8 | 21.8 | 26.8 | 24.8 | 22.7 | 23.7 |
| *P. multocida* | OF |  |  |  |  |  |  |  |  | 29.9 | 29.8 |  |  |  |  |  |  | 27.9 |  |
| *B. pilosicoli* | FS |  |  |  |  |  |  |  |  |  |  |  |  |  |  |  |  |  |  |
| *L. intracellularis* | FS |  |  |  |  | 19.2 | 21.1 |  |  |  |  | 25.0 | 22.8 |  |  |  |  | 17.0 |  |
| *E. coli* F4 | FS |  | 23.5 |  |  |  | 27.4 |  | 17.6 |  |  |  |  | 20.6 | 19.9 |  |  |  |  |
| *E. coli* F18 | FS | 18.2 | 21.2 |  |  |  |  | 20.8 | 15.6 |  |  |  |  | 16.4 | 15.5 |  |  |  |  |
| PCV3 | FS |  |  |  |  |  |  |  |  |  |  |  |  | 23.5 | 26.7 |  |  |  |  |
| PCV2 | FS |  |  |  |  |  |  |  |  | 26.9 |  |  |  |  |  |  |  | 27.5 |  |
| Rotavirus A | FS | 27.9 | 27.4 |  |  | 30.5 |  | 21.2 | 17.4 |  | 22.9 |  |  | 17.7 | 17.1 | 24.6 | 21.5 | 29.7 |  |

| **B) 1F – oral fluid (OF) and faecal sock (FS) samples** | | | | | | | | | | | | | |
| --- | --- | --- | --- | --- | --- | --- | --- | --- | --- | --- | --- | --- | --- |
| Sampling date | | September | | | | October | | | | November | | | |
| Days after achieving a weight of 30 kg | | ? (45-80 kg) | | ? (80-100 kg) | | ? (45-60 kg) | | ? (50-85 kg) | | ? (65 kg) | | ? (75-80 kg) | |
| Pathogen | Material | **Pen 1** | **Pen 2** | **Pen 1** | Pen 2 | Pen 1 | Pen 2 | **Pen 1** | Pen 2 | **Pen 1** | **Pen 2** | **Pen 1** | **Pen 2** |
| swIAV | OF |  |  |  |  |  |  |  | 24.9 | 20.3 | 27.1 |  |  |
| A(H1)pdm09 | OF |  |  |  |  |  |  |  |  |  |  |  |  |
| PRRSV-1 | OF |  |  |  |  |  |  |  |  |  |  |  |  |
| PRRSV-2 | OF |  |  |  |  |  |  |  |  |  |  |  |  |
| PCMV | OF | 21.3 | 24.8 | 21.7 | 22.5 | 24.6 | 18.0 | 21.7 | 24.3 | 22.2 | 18.7 | 20.5 | 18.8 |
| *A. pleuropneumoniae* | OF |  |  |  | 23.9 | 24.3 | 22.9 | 24.8 | 22.2 | 22.9 | 22.9 | 23.1 | 22.8 |
| PCV2 | OF |  | 24.7 |  | 25.0 |  |  |  |  |  |  |  | 25.7 |
| *S. suis* type 2 | OF | 22.5 | 22.9 | 22.1 | 21.3 | 24.6 | 22.7 | 22.0 | 21.5 | 19.8 | 20.2 | 20.6 | 21.1 |
| *B. bronchiseptica* | OF |  |  |  |  |  |  |  |  |  |  |  |  |
| PCV3 | OF |  |  | 27.1 | 26.3 |  |  |  | 27.5 | 27.3 | 30.2 |  | 31.4 |
| PPV | OF |  |  |  |  |  |  |  |  |  |  |  |  |
| *M. hyopneumoniae* | OF |  |  |  |  |  |  |  |  |  |  |  |  |
| *M. hyorhinis* | OF | 26.0 | 28.8 | 25.1 | 26.5 | 27.4 | 26.0 | 26.6 | 27.9 | 19.6 | 21.8 | 23.5 | 25.2 |
| *P. multocida* | OF |  |  |  |  | 30.4 |  |  | 29.2 |  |  |  |  |
| *B. pilosicoli* | FS |  |  |  |  |  |  |  |  |  |  |  |  |
| *L. intracellularis* | FS |  |  |  |  |  | 21.8 | 25.0 |  |  |  |  |  |
| *E. coli* F4 | FS |  |  |  |  |  |  |  |  |  |  |  |  |
| *E. coli* F18 | FS |  |  |  |  | 24.6 | 24.7 | 24.8 |  |  |  |  |  |
| PCV3 | FS |  |  |  |  |  |  |  |  |  |  |  |  |
| PCV2 | FS |  |  |  |  |  |  |  |  |  |  |  |  |
| Rotavirus A | FS |  | 26.4 |  |  | 24.5 | 31.4 |  | 27.1 |  |  |  |  |

| **C) 2N – oral fluid (OF) and faecal sock (FS) samples** | | | | | | | | | | | | | | | | | | | |
| --- | --- | --- | --- | --- | --- | --- | --- | --- | --- | --- | --- | --- | --- | --- | --- | --- | --- | --- | --- |
| Sampling date | | September | | | | | | October | | | | | | November | | | | | |
| Days after weaning | | 9 | | 37 | | 65 | | 10 | | 24 | | 38 | | 2 | | 29-36 | | 48 | |
| Pathogen | Material | ***Pen 1*** | ***Pen 2*** | Pen 1 | Pen 2 | **Pen 1** | **Pen 2** | Pen 1 | ***Pen 2*** | Pen 1 | Pen 2 | Pen 1 | **Pen 2** | Pen 1 | Pen 2 | Pen 1 | **Pen 2** | Pen 1 | Pen 2 |
| swIAV | OF | 14.9 | 14.2 |  | 18.6 |  |  | 12.8 | 12.2 | 15.4 | 14.9 | 22.7 | 27.6 | na | na |  | 15.7 | 25.6 | 24.0 |
| A(H1)pdm09 | OF |  |  |  |  |  |  |  |  |  |  |  |  | na | na |  |  |  |  |
| PRRSV-1 | OF |  |  |  |  |  |  |  |  |  |  |  |  | na | na |  |  |  |  |
| PRRSV-2 | OF |  |  |  |  |  |  |  |  |  |  |  |  | na | na |  |  |  |  |
| PCMV | OF | 8.0 | 10.7 | 18.7 | 18.0 |  | 20.6 | 11.2 | 10.2 | 13.1 | 13.4 | 19.5 | 18.2 | na | na | 17.8 | 18.8 | 22.8 | 17.9 |
| *A. pleuropneumoniae* | OF |  |  |  |  |  |  | 24.0 | 24.3 | 24.4 | 25.2 | 24.7 |  | na | na |  | 23.4 |  |  |
| PCV2 | OF | 24.6 |  | 18.7 | 20.3 | 14.9 | 14.6 |  |  |  |  | 26.9 |  | na | na | 26.0 |  |  | 21.8 |
| *S. suis* type 2 | OF | 23.4 | 23.2 | 21.8 | 23.3 | 23.6 | 22.3 | 21.3 | 21.8 | 19.7 | 21.6 | 22.0 | 21.4 | na | na | 21.2 | 21.9 | 23.3 | 23.8 |
| *B. bronchiseptica* | OF |  |  |  | 25.7 |  |  |  |  |  |  | 26.6 | 25.8 | na | na | 25.6 |  |  |  |
| PCV3 | OF |  |  | 26.6 | 23.7 | 19.5 | 26.8 |  |  | 20.4 | 22.8 |  |  | na | na | 22.3 | 27.6 | 29.7 | 28.8 |
| PPV | OF | 26.2 | 27.4 |  | 25.76 | 18.7 | 17.9 | 28.4 | 30.3 |  |  | 27.1 | 30.7 | na | na |  | 32.3 |  |  |
| *M. hyopneumoniae* | OF |  |  |  |  |  |  |  |  |  |  |  |  | na | na |  |  |  |  |
| *M. hyorhinis* | OF | 26.3 | 25.9 | 26.3 |  | 23.9 |  | 25.8 | 23.2 | 21.4 | 23.2 | 24.7 | 24.5 | na | na | 22.0 | 24.9 | 24.7 |  |
| *P. multocida* | OF |  |  |  |  |  |  | 25.4 |  | 19.4 |  |  |  | na | na |  | 34.8 |  |  |
| *B. pilosicoli* | FS |  |  |  |  |  |  |  |  |  |  |  |  |  |  | 21.6 |  |  |  |
| *L. intracellularis* | FS |  |  | 21.6 | 20.5 | 20.7 | 16.8 |  |  | 24.9 | 26.2 | 22.4 | 15.4 |  |  | 12.1 | 10.2 | 19.0 | 18.8 |
| *E. coli* F4 | FS | 20.7 | 24.1 |  |  |  |  | 21.9 | 22.0 |  |  |  |  | 14.6 | 15.3 |  |  |  |  |
| *E. coli* F18 | FS | 22.8 |  |  |  |  |  | 24.0 | 26.6 | 24.2 |  |  |  |  |  |  |  |  |  |
| PCV3 | FS |  |  |  |  | 27.9 |  |  |  |  |  |  |  | 24.9 | 23.1 |  |  |  |  |
| PCV2 | FS |  |  |  |  | 15.7 | 16.2 |  |  |  |  |  |  |  |  |  |  |  |  |
| Rotavirus A | FS | 26.1 | 19.1 | 30.5 | 32.9 | 29.7 |  | 22.9 | 21.2 | 21.0 | 17.9 | 29.7 |  | 13.5 | 13.1 | 24.2 | 23.2 | 21.0 | 15.7 |

na: no analysis

| **D) 3N – oral fluid (OF) and faecal sock (FS) samples** | | | | | | | | | | | | | | | | | | | |
| --- | --- | --- | --- | --- | --- | --- | --- | --- | --- | --- | --- | --- | --- | --- | --- | --- | --- | --- | --- |
| Sampling date | | September | | | | | | October | | | | | | November | | | | | |
| Days after weaning | | 10 | | 24 | | 38 | | 12 | | 26 | | 40 | | 16 | | 30 | | 45 | |
| Pathogen | Material | Pen 1 | Pen 2 | Pen 1 | Pen 2 | **Pen 1** | **Pen 2** | Pen 1 | Pen 2 | Pen 1 | Pen 2 | Pen 1 | Pen 2 | Pen 1 | ***Pen 2*** | ***Pen 1*** | Pen 2 | **Pen 1** | **Pen 2** |
| swIAV | OF | 13.0 | 24.0 |  |  | 26.1 |  | 13.5 | 14.9 |  |  |  |  |  |  | 22.5 | 13.2 | 11.7 | 21.4 |
| A(H1)pdm09 | OF |  |  |  |  |  |  |  |  |  |  |  |  |  |  |  |  |  |  |
| PRRSV-1 | OF |  |  |  |  |  |  |  |  |  |  |  |  |  |  |  |  |  |  |
| PRRSV-2 | OF |  |  |  |  |  |  |  |  |  |  |  |  |  |  |  |  |  |  |
| PCMV | OF | 14.2 | 14.0 | 17.5 | 11.3 | 16.7 | 20.1 | 15.0 | 11.5 | 12.2 | 14.4 | 18.2 | 17.3 | 11.4 | 10.9 | 10.8 | 14.0 | 20.2 | 21.4 |
| *A. pleuropneumoniae* | OF |  |  |  |  |  |  |  |  |  |  |  |  |  |  |  |  |  |  |
| PCV2 | OF |  |  |  |  |  |  |  |  |  |  |  |  | 23.4 |  | 25.3 |  |  |  |
| *S. suis* type 2 | OF | 20.0 | 24.1 | 24.2 | 22.3 | 24.3 | 22.6 | 18.9 | 19.4 | 21.4 | 22.6 | 20.7 | 19.5 | 21.8 | 22.6 | 22.4 | 24.5 | 21.8 | 22.3 |
| *B. bronchiseptica* | OF | 23.1 |  |  |  | 26.3 |  | 25.6 | 28.5 | 27.5 |  | 26.3 |  | 28.1 |  |  | 27.7 |  |  |
| PCV3 | OF | 16.4 | 26.8 | 26.7 | 27.4 | 25.9 |  | 28.9 | 19.6 | 27.6 | 21.9 | 21.9 | 26.4 |  | 26.2 | 25.8 |  | 24.2 | 25.0 |
| PPV | OF |  |  |  |  |  |  |  |  |  |  |  |  |  |  |  |  |  |  |
| *M. hyopneumoniae* | OF |  |  |  |  |  |  |  |  |  |  |  |  |  |  |  |  |  |  |
| *M. hyorhinis* | OF |  |  | 25.6 | 21.8 | 23.7 | 22.9 | 25.2 |  |  |  | 22.6 | 21.1 | 17.9 | 20.9 | 22.0 | 23.0 | 24.9 | 21.1 |
| *P. multocida* | OF |  |  |  |  |  |  | 24.9 | 26.6 | 27.9 | 30.0 |  |  |  |  |  |  |  |  |
| *B. pilosicoli* | FS |  |  |  |  |  |  |  | 21.6 | 17.2 | 23.3 |  | 26.5 |  |  | 18.6 | 19.6 |  | 19.3 |
| *L. intracellularis* | FS |  |  |  |  | 23.6 |  |  |  |  | 21.4 | 16.9 |  | 21.1 |  | 26.8 |  | 15.8 | 15.3 |
| *E. coli* F4 | FS | 22.4 |  |  |  |  |  |  |  |  |  |  | 17.2 |  |  |  |  | 25.4 |  |
| *E. coli* F18 | FS | 15.9 | 19.8 |  | 24.8 |  |  |  | 25.3 | 20.9 |  |  | 21.2 | 22.1 |  | 27.7 |  |  | 18.9 |
| PCV3 | FS | 24.0 | 24.3 |  |  |  |  |  | 25.3 |  |  | 22.3 |  |  |  |  |  |  |  |
| PCV2 | FS |  | 25.7 |  |  |  |  |  |  |  |  |  |  |  |  |  |  |  |  |
| Rotavirus A | FS | 24.0 | 22.9 |  | 25.4 |  |  | 19.4 | 21.0 | 26.4 | 23.7 |  |  | 19.4 | 23.0 | 25.7 |  |  |  |

| **E) 3F – oral fluid (OF) and faecal sock (FS) samples** | | | | | | | | | | | | | | | | | | | |
| --- | --- | --- | --- | --- | --- | --- | --- | --- | --- | --- | --- | --- | --- | --- | --- | --- | --- | --- | --- |
| Sampling date | | September | | | | | | October | | | | | | November | | | | | |
| Days after achieving a weight of 30 kg | | 15 | | 35 | | 70 | | 14 | | 44 | | 64 | | 16 | | 46 | | 70 | |
| Pathogen | Material | **Pen 1** | Pen 2 | **Pen 1** | Pen 2 | **Pen 1** | **Pen 2** | Pen 1 | Pen 2 | **Pen 1** | Pen 2 | **Pen 1** | Pen 2 | Pen 1 | **Pen 2** | Pen 1 | Pen 2 | Pen 1 | **Pen 2** |
| swIAV | OF |  | 25.7 |  | 24.2 |  |  | 18.3 |  |  |  |  |  | 22.8 |  |  |  |  |  |
| A(H1)pdm09 | OF |  |  |  |  |  |  |  |  |  |  |  |  |  |  |  |  |  |  |
| PRRSV-1 | OF |  |  |  |  |  |  |  |  |  |  |  |  |  |  |  |  |  |  |
| PRRSV-2 | OF |  |  |  |  |  |  |  |  |  |  |  |  |  |  |  |  |  |  |
| PCMV | OF | 22.8 | 20.8 | 19.0 | 22.5 |  |  | 20.3 | 17.0 |  | 21.9 | 20.2 | 21.6 | 23.7 | 22.8 | 20.4 |  | 21.0 |  |
| *A. pleuropneumoniae* | OF |  |  |  |  |  |  |  |  |  |  |  |  |  |  |  |  |  |  |
| PCV2 | OF | 20.2 |  |  |  |  |  | 21.8 | 26.0 |  | 16.8 |  |  | 19.7 | 23.1 |  | 28.5 |  |  |
| *S. suis* type 2 | OF | 22.4 | 21.9 | 21.6 | 20.4 | 23.9 | 25.1 | 21.2 | 21.9 | 23.4 | 23.9 | 21.5 | 21.3 | 24.2 | 25.8 | 27.7 | 23.3 | 21.4 | 24.3 |
| *B. bronchiseptica* | OF |  |  |  |  |  |  | 26.0 |  |  |  |  |  | 26.7 |  |  |  |  |  |
| PCV3 | OF | 16.5 | 18.5 | 26.4 | 20.0 | 24.9 |  | 25.2 |  | 29.0 | 22.8 | 17.0 | 24.4 | 30.8 | 28.8 |  | 28.5 | 21.2 |  |
| PPV | OF | 24.5 | 27.0 |  |  |  |  | 27.1 |  |  |  |  |  | 30.4 | 27.2 |  |  |  |  |
| *M. hyopneumoniae* | OF |  |  |  |  |  |  |  |  |  |  |  |  |  |  |  |  |  |  |
| *M. hyorhinis* | OF | 24.5 | 23.6 | 25.7 | 25.1 |  |  | 20.2 | 21.6 |  | 27.9 |  | 25.2 | 20.6 | 22.9 |  |  | 26.0 |  |
| *P. multocida* | OF |  |  |  |  |  |  |  |  |  |  |  |  |  |  |  |  |  |  |
| *B. pilosicoli* | FS |  |  | 21.4 |  | 20.0 | 15.9 |  | 25.0 |  |  | 23.6 | 23.4 |  |  | 17.5 | 22.0 |  | 20.5 |
| *L. intracellularis* | FS | 17.6 |  |  | 25.6 |  |  | 23.2 | 18.1 |  |  |  |  |  | 18.1 | 26.7 |  |  |  |
| *E. coli* F4 | FS |  |  |  |  |  |  |  |  |  |  |  |  |  |  |  |  |  |  |
| *E. coli* F18 | FS |  | 19.2 |  |  |  |  |  |  |  |  |  |  |  | 20.6 | 31.0 |  |  |  |
| PCV3 | FS | 25.2 | 24.9 | 27.7 | 23.9 | 29.1 | 27.9 |  |  | 29.5 | 28.6 | 27.2 |  |  |  |  |  |  |  |
| PCV2 | FS |  |  |  |  |  | 25.5 | 27.9 |  |  |  |  |  | 24.2 |  | 27.5 | 27.5 |  |  |
| Rotavirus A | FS |  | 22.4 | 29.4 |  |  | 24.9 |  | 20.7 |  |  |  |  | 26.0 | 31.6 |  |  |  |  |

| **F) 4N – oral fluid (OF) and faecal sock (FS) samples** | | | | | | | | | | | | | | | | | | | |
| --- | --- | --- | --- | --- | --- | --- | --- | --- | --- | --- | --- | --- | --- | --- | --- | --- | --- | --- | --- |
| Sampling date | | September | | | | | | October | | | | | | November | | | | | |
| Days after weaning | | 17 | | 31 | | 45 | | 17 | | 31 | | 45 | | 20 | | 34 | | 48 | |
| Pathogen | Material | Pen 1 | **Pen 2** | Pen 1 | Pen 2 | Pen 1 | Pen 2 | Pen 1 | Pen 2 | Pen 1 | Pen 2 | **Pen 1** | Pen 2 | **Pen 1** | **Pen 2** | **Pen 1** | **Pen 2** | **Pen 1** | **Pen 2** |
| swIAV | OF |  |  |  | 25.5 |  |  |  |  |  |  |  |  |  |  |  |  |  |  |
| A(H1)pdm09 | OF |  |  |  |  |  |  |  |  |  |  |  |  |  |  |  |  |  |  |
| PRRSV-1 | OF |  |  |  |  |  |  |  |  |  |  |  |  |  |  |  |  |  |  |
| PRRSV-2 | OF |  |  |  |  |  |  |  |  |  |  |  |  |  |  |  |  |  |  |
| PCMV | OF | 13.2 | 17.8 | 15.0 | 11.5 | 18.3 | 17.6 | 19.8 | 17.1 | 13.3 | 12.4 | 15.6 | 18.2 | 16.1 | 16.0 | 16.3 | 13.7 | 20.8 | 21.0 |
| *A. pleuropneumoniae* | OF |  |  |  |  |  |  |  |  |  |  |  |  |  |  |  |  |  |  |
| PCV2 | OF | 28.4 | 26.2 |  | 24.6 |  |  | 24.6 | 25.8 | 28.0 | 24.7 |  | 27.6 | 26.0 |  |  | 33.4 | 22.8 | 24.6 |
| *S. suis* type 2 | OF | 19.4 | 22.2 | 21.2 | 22.1 | 20.2 | 22.0 | 19.9 | 20.6 | 20.8 | 22.4 | 20.8 | 20.0 | 22.3 | 22.1 | 20.6 | 20.2 | 21.1 | 21.8 |
| *B. bronchiseptica* | OF | 25.5 | 26.9 | 25.5 | 24.7 | 28.1 | 26.3 |  |  | 24.8 |  |  |  |  |  |  |  |  | 28.4 |
| PCV3 | OF |  |  |  |  |  |  | 27.3 |  | 24.7 |  | 27.5 |  |  |  | 24.0 |  | 25.9 | 31.0 |
| PPV | OF |  |  |  |  |  |  |  |  |  |  |  |  |  |  |  |  |  |  |
| *M. hyopneumoniae* | OF |  |  |  |  |  |  |  |  |  |  |  |  |  |  |  |  |  |  |
| *M. hyorhinis* | OF | 22.5 | 24.6 | 25.1 |  |  |  | 19.6 | 20.8 | 23.7 | 24.5 | 27.5 | 25.1 |  | 23.4 | 25.3 |  | 23.4 | 27.1 |
| *P. multocida* | OF |  |  |  |  |  |  | 27.9 |  |  |  |  |  |  |  |  |  |  |  |
| *B. pilosicoli* | FS |  |  |  | 17.7 |  |  | 27.2 | 24.9 |  | 25.0 | 17.5 | 17.6 |  |  | 18.1 | 14.7 | 16.0 | 15.5 |
| *L. intracellularis* | FS |  |  |  |  |  | 25.2 |  |  |  |  |  |  |  |  |  |  | 16.9 | 21.4 |
| *E. coli* F4 | FS | 23.8 | 25.6 | 19.9 | 23.6 |  |  | 21.9 |  |  |  |  |  | 17.3 | 17.2 | 27.4 |  |  |  |
| *E. coli* F18 | FS | 14.1 | 18.3 |  |  |  |  | 14.1 | 22.0 | 18.3 |  |  |  | 16.4 | 14.3 |  |  |  |  |
| PCV3 | FS | 26.5 |  |  |  |  |  |  |  |  |  |  |  |  |  |  |  |  |  |
| PCV2 | FS | 27.2 |  |  |  |  |  | 26.7 |  |  | 28.3 |  |  |  |  |  |  |  |  |
| Rotavirus A | FS | 24.5 | 22.5 | 27.4 | 31.2 | 25.4 | 26.3 | 28.4 | 25.4 | 23.9 |  |  |  | 31.4 |  |  |  |  |  |

| **G) 4F – oral fluid (OF) and faecal sock (FS) samples** | | | | | | | | | | | | | | | | | | | |
| --- | --- | --- | --- | --- | --- | --- | --- | --- | --- | --- | --- | --- | --- | --- | --- | --- | --- | --- | --- |
| Sampling date | | September | | | | | | October | | | | | | November | | | | | |
| Days after achieving a weight of 30 kg | | 10 | | 38 | | 74 | | 24 | | 38 | | 66 | | 13 | | 24 | | 69 | |
| Pathogen | Material | Pen 1 | **Pen 2** | **Pen 1** | Pen 2 | Pen 1 | Pen 2 | Pen 1 | **Pen 2** | **Pen 1** | **Pen 2** | **Pen 1** | Pen 2 | Pen 1 | Pen 2 | Pen 1 | **Pen 2** | Pen 1 | Pen 2 |
| swIAV | OF |  |  |  |  |  | 24.4 |  |  |  |  |  |  |  |  |  |  |  |  |
| A(H1)pdm09 | OF |  |  |  |  |  |  |  |  |  |  |  |  |  |  |  |  |  |  |
| PRRSV-1 | OF |  |  |  |  |  |  |  |  |  |  |  |  |  |  |  |  |  |  |
| PRRSV-2 | OF |  |  |  |  |  |  |  |  |  |  |  |  |  |  |  |  |  |  |
| PCMV | OF | 17.0 | 18.4 | 22.3 | 21.6 |  |  | 22.0 | 19.3 | 26.4 |  | 23.7 | 23.2 | 19.8 | 18.3 | 20.4 | 22.0 | 23.6 | 20.1 |
| *A. pleuropneumoniae* | OF |  |  |  |  |  |  |  |  |  |  |  |  |  |  |  |  |  |  |
| PCV2 | OF | 22.4 | 23.7 | 6.9 | 9.3 | 15.5 | 15.8 | 11.7 | 12.7 | 10.4 | 27.7 | 15.9 | 13.6 | 12.7 | 28.0 | 21.7 |  | 14.9 | 14.6 |
| *S. suis* type 2 | OF | 22.3 | 22.0 | 23.7 | 23.4 | 23.5 | 21.5 | 29.5 | 24.8 | 24.4 |  | 21.8 | 21.5 | 20.4 | 22.1 | 23.0 | 24.8 | 25.4 | 20.9 |
| *B. bronchiseptica* | OF |  |  |  |  |  |  |  |  |  |  |  |  | 25.7 | 27.6 |  |  |  |  |
| PCV3 | OF |  |  |  | 24.7 |  | 25.5 |  |  | 19.6 |  |  | 23.0 | 26.9 |  |  |  |  |  |
| PPV | OF | 27.3 | 28.0 |  | 27.7 |  |  | 29.1 |  | 16.8 | 23.5 | 23.3 | 25.0 |  |  |  |  | 21.2 | 25.9 |
| *M. hyopneumoniae* | OF |  |  |  |  |  |  |  |  |  |  |  |  |  |  |  |  |  |  |
| *M. hyorhinis* | OF | 27.9 | 26.8 |  |  |  |  |  | 25.7 | 26.4 |  | 28.6 |  | 24.4 |  |  |  |  |  |
| *P. multocida* | OF | 25.7 | 23.7 |  |  |  |  |  |  |  |  |  |  |  |  |  |  |  |  |
| *B. pilosicoli* | FS |  | 24.0 | 19.6 | 21.7 |  | 18.9 |  |  | 18.6 | 18.4 | 18.5 | 19.7 | 25.1 | 23.9 |  |  | 21.1 | 25.4 |
| *L. intracellularis* | FS | 24.5 | 16.9 | 17.9 |  |  | 25.3 | 18.4 | 14.3 | 21.2 | 26.6 | 24.0 | 27.2 | 15.7 | 15.0 | 22.2 | 16.9 |  |  |
| *E. coli* F4 | FS |  |  |  |  |  |  |  |  |  |  |  | 26.6 |  |  |  |  |  |  |
| *E. coli* F18 | FS |  |  |  |  |  |  |  |  |  |  |  |  |  |  |  |  |  |  |
| PCV3 | FS |  |  |  | 24.7 |  | 25.5 |  |  | 24.8 |  |  |  |  |  |  |  |  |  |
| PCV2 | FS | 20.5 | 25.2 | 8.1 | 9.9 | 18.9 | 19.8 | 11.3 | 15.2 | 12.1 | 17.1 | 16.5 | 16.2 | 12.6 |  | 29.5 |  | 17.4 | 17.0 |
| Rotavirus A | FS | 22.0 |  |  |  | 27.8 | 27.2 |  |  |  |  |  |  |  |  |  |  |  |  |

| **H) 5N – oral fluid (OF) and faecal sock (FS) samples** | | | | | | | | | | | | | | | | | | | |
| --- | --- | --- | --- | --- | --- | --- | --- | --- | --- | --- | --- | --- | --- | --- | --- | --- | --- | --- | --- |
| Sampling date | | September | | | | | | October | | | | | | November | | | | | |
| Days after weaning | | 12 | | 33 | | 46 | | 12 | | 33 | | 46 | | 15 | | 29 | | 43 | |
| Pathogen | Material | Pen 1 | Pen 2 | Pen 1 | Pen 2 | Pen 1 | Pen 2 | Pen 1 | Pen 2 | Pen 1 | ***Pen 2*** | Pen 1 | Pen 2 | Pen 1 | Pen 2 | Pen 1 | Pen 2 | Pen 1 | **Pen 2** |
| swIAV | OF | 11.6 | 10.4 |  | 21.0 | 28.0 |  | 17.0 | 15.8 |  | 20.1 |  | 25.6 | 12.1 | 10.6 |  |  |  |  |
| A(H1)pdm09 | OF |  |  |  |  |  |  |  |  |  |  |  |  |  |  |  |  |  |  |
| PRRSV-1 | OF |  |  |  |  |  |  |  |  |  |  |  |  |  |  |  |  |  |  |
| PRRSV-2 | OF |  |  |  |  |  |  |  |  |  |  |  |  |  |  |  |  |  |  |
| PCMV | OF | 18.9 | 15.3 | 12.0 | 12.4 | 21.8 | 21.2 | 14.0 | 15.9 | 16.4 | 13.6 | 21.0 | 20.8 | 11.1 | 12.2 | 11.1 | 12.1 | 19.0 | 18.3 |
| *A. pleuropneumoniae* | OF |  |  |  |  |  |  |  |  |  |  |  |  |  |  |  |  |  |  |
| PCV2 | OF |  |  | 24.9 | 20.9 | 21.4 | 23.2 | 19.9 | 11.5 |  | 23.8 | 17.8 | 19.2 | 21.5 | 23.1 | 17.8 | 13.6 | 19.6 | 15.9 |
| *S. suis* type 2 | OF | 20.1 | 23.2 | 23.5 | 22.3 | 23.9 | 21.7 | 20.3 | 23.3 | 22.0 | 21.8 | 22.5 | 22.5 | 19.1 | 19.2 | 18.9 | 21.5 | 21.5 | 21.2 |
| *B. bronchiseptica* | OF |  |  |  | 28.6 |  | 27.3 | 28.0 | 25.4 | 27.2 |  |  | 25.9 | 25.9 | 24.3 | 24.5 |  | 28.2 |  |
| PCV3 | OF |  |  | 28.8 | 22.6 | 24.9 | 21.2 |  | 28.3 |  | 26.3 |  | 28.9 | 17.2 |  | 20.8 |  | 21.6 | 16.6 |
| PPV | OF |  |  |  |  |  |  |  |  |  |  |  |  |  |  |  |  |  |  |
| *M. hyopneumoniae* | OF |  |  |  |  |  |  |  |  |  |  |  |  |  |  |  |  |  |  |
| *M. hyorhinis* | OF | 23.6 | 25.5 | 21.9 | 24.0 | 27.8 | 25.5 | 19.6 | 21.4 | 22.8 | 23.0 | 27.4 | 23.3 | 19.0 | 18.7 | 20.4 | 20.6 | 21.7 | 21.8 |
| *P. multocida* | OF |  |  |  |  |  |  |  |  |  |  |  | 26.9 | 28.7 | 32.3 |  |  |  |  |
| *B. pilosicoli* | FS |  |  |  |  |  |  |  |  |  |  |  |  |  | 20.3 |  |  |  | 13.4 |
| *L. intracellularis* | FS |  |  |  |  | 19.7 | 25.0 |  |  |  |  | 11.8 | 20.5 |  |  |  |  | 18.6 | 25.7 |
| *E. coli* F4 | FS |  |  | 24.7 |  |  |  | 29.9 | 23.9 |  |  |  |  | 16.7 |  |  |  |  |  |
| *E. coli* F18 | FS | 25.2 | 23.6 |  |  | 27.6 |  |  | 17.4 | 15.9 | 29.4 |  |  | 18.9 | 21.1 | 26.6 |  |  |  |
| PCV3 | FS |  |  |  |  |  |  |  |  |  |  |  |  | 25.8 |  |  |  |  | 28.7 |
| PCV2 | FS |  |  | 25.9 |  | 25.8 | 19.8 | 21.3 | 12.5 |  |  | 16.1 | 21.2 | 24.3 | 22.6 | 21.1 | 16.2 | 23.2 | 16.0 |
| Rotavirus A | FS | 18.4 | 22.5 |  | 22.4 | 28.2 |  | 19.8 | 24.0 | 26.8 | 24.0 | 27.5 | 21.7 | 19.3 | 13.0 | 27.7 | 24.3 | 24.8 | 23.1 |

| **I) 6N – oral fluid (OF) and faecal sock (FS) samples** | | | | | | | | | | | | | | | | | | | | |
| --- | --- | --- | --- | --- | --- | --- | --- | --- | --- | --- | --- | --- | --- | --- | --- | --- | --- | --- | --- | --- |
| Sampling date | | September | | | | | | October | | | | | | November | | | | | | |
| Days after weaning | | 17 | | 31 | | 45 | | 17 | | 31 | | 52 | | 15 | | 29 | | | 50 | |
| Pathogen | Material | **Pen 1** | **Pen 2** | Pen 1 | **Pen 2** | **Pen 1** | **Pen 2** | **Pen 1** | **Pen 2** | **Pen 1** | Pen 2 | **Pen 1** | Pen 2 | **Pen 1** | **Pen 2** | **Pen 1** | **Pen 2** | ***Pen 2*** | **Pen 1** | **Pen 2** |
| swIAV | OF |  | 22.1 | 14.7 |  | 14.1 |  |  | 18.4 |  |  |  | 27.2 |  | 22.9 | 15.9 |  | |  |  |
| A(H1)pdm09 | OF |  |  |  |  | 25.2 |  |  |  |  |  |  |  |  |  |  |  | |  |  |
| PRRSV-1 | OF |  |  |  |  |  |  |  |  |  |  |  |  |  |  |  |  | |  |  |
| PRRSV-2 | OF |  |  |  |  |  |  |  |  |  |  |  |  |  |  |  |  | |  |  |
| PCMV | OF | 10.8 | 16.2 | 19.0 |  | 21.8 | 19.6 | 14.7 | 10.7 | 14.6 | 11.3 | 20.7 | 23.0 | 11.0 | 12.8 | 12.7 | 13.2 | |  | 18.4 |
| *A. pleuropneumoniae* | OF |  |  |  |  |  |  |  |  |  |  |  |  |  |  |  | 24.9 | |  |  |
| PCV2 | OF | 21.9 | 21.8 | 16.4 |  | 8.5 | 18.1 | 22.1 | 20.6 | 22.1 | 22.7 | 15.8 | 9.1 | 19.7 | 20.2 | 24.2 | 23.0 | | 15.9 | 15.5 |
| *S. suis* type 2 | OF | 25.4 | 24.5 | 22.7 |  | 22.5 | 22.0 | 23.8 | 23.3 | 22.8 | 22.3 | 20.4 | 22.2 | 19.8 | 21.5 | 21.7 | 22.0 | | 22.7 | 21.8 |
| *B. bronchiseptica* | OF |  |  |  |  |  | 26.1 |  |  | 25.8 |  | 26.2 |  | 27.8 | 27.7 | 26.2 |  | |  |  |
| PCV3 | OF | 29.3 |  | 26.5 |  |  | 28.2 |  | 27.4 |  |  | 27.0 |  | 25.0 |  | 24.9 | 27.7 | | 27.6 |  |
| PPV | OF |  |  |  |  | 24.1 |  |  |  |  |  |  |  |  |  |  |  | |  |  |
| *M. hyopneumoniae* | OF |  |  |  |  |  |  |  |  |  |  |  |  |  |  |  |  | |  |  |
| *M. hyorhinis* | OF |  |  |  |  |  |  |  |  |  |  |  |  |  |  |  |  | |  |  |
| *P. multocida* | OF |  |  |  |  |  |  | 27.4 |  | 27.3 |  | 30.1 |  | 24.4 |  | 26.2 |  | |  |  |
| *B. pilosicoli* | FS |  |  | 25.1 | 23.1 | 20.0 |  |  |  | 25.8 | 21.5 | 15.1 | 14.7 |  |  |  | 20.8 | | 16.2 | 21.2 |
| *L. intracellularis* | FS |  |  | 17.1 | 18.6 | 18.6 | 24.3 |  | 14.6 |  | 21.2 | 13.7 | 19.7 |  |  | 24.9 | 19.9 | | 14.4 | 15.3 |
| *E. coli* F4 | FS |  |  |  |  |  |  |  |  |  |  |  |  | 20.4 | 21.8 | 27.3 |  | |  |  |
| *E. coli* F18 | FS | 20.8 | 23.3 |  |  |  |  | 22.0 | 25.1 |  |  |  |  | 19.9 | 22.1 |  |  | |  |  |
| PCV3 | FS |  |  |  |  |  |  |  |  |  |  |  |  |  |  |  | 30.3 | |  |  |
| PCV2 | FS |  |  | 17.2 | 13.1 | 13.6 | 19.5 | 26.7 | 20.1 |  |  | 18.8 | 11.6 |  |  |  |  | | 19.0 | 20.3 |
| Rotavirus A | FS | 28.6 | 26.0 | 24.1 | 30.1 |  |  | 16.0 | 23.6 | 20.6 | 24.9 |  |  | 18.7 | 17.3 | 23.7 |  | | 22.5 |  |

| **J) 6F – oral fluid (OF) and faecal sock (FS) samples** | | | | | | | | | | | | | | | | | | | |
| --- | --- | --- | --- | --- | --- | --- | --- | --- | --- | --- | --- | --- | --- | --- | --- | --- | --- | --- | --- |
| Sampling date | | September | | | | | | October | | | | | | November | | | | | |
| Days after achieving a weight of 30 kg | | 14 | | 30 | | 56 | | 14 | | 42 | | 62 | | 19 | | 40 | | 68 | |
| Pathogen | Material | **Pen 1** | **Pen 2** | Pen 1 | Pen 2 | **Pen 1** | **Pen 2** | **Pen 1** | **Pen 2** | Pen 1 | **Pen 2** | Pen 1 | Pen 2 | **Pen 1** | **Pen 2** | **Pen 1** | **Pen 2** | **Pen 1** | **Pen 2** |
| swIAV | OF |  |  |  |  |  |  | 21.5 |  |  |  |  |  | 21.7 | 26.9 |  |  |  |  |
| A(H1)pdm09 | OF |  |  |  |  |  |  |  |  |  |  |  |  |  |  |  |  |  |  |
| PRRSV-1 | OF |  |  |  |  |  |  |  |  |  |  |  |  |  |  |  |  |  |  |
| PRRSV-2 | OF |  |  |  |  |  |  |  |  |  |  |  |  |  |  |  |  |  |  |
| PCMV | OF | 21.4 | 23.1 | 26.3 | 24.1 |  |  | 23.3 | 21.3 | 21.3 | 22.1 | 24.1 | 21.5 | 21.4 | 23.1 | 21.0 | 23.3 | 25.6 | 26.9 |
| *A. pleuropneumoniae* | OF | 25.4 | 26.9 | 25.5 | 24.1 | 24.6 | 25.6 |  |  | 23.7 |  | 24.5 | 23.0 |  | 24.9 |  |  | 25.4 | 22.9 |
| PCV2 | OF | 13.7 | 14.4 | 14.4 | 24.4 | 19.5 | 20.3 | 11.5 | 11.9 | 12.6 | 19.0 | 16.4 | 15.6 | 9.0 | 8.5 | 18.5 | 18.0 | 17.4 | 13.0 |
| *S. suis* type 2 | OF | 26.1 | 24.6 | 21.1 | 22.7 | 23.6 | 23.9 | 22.4 | 22.0 | 23.0 | 23.6 | 20.7 | 19.9 | 21.0 | 21.1 | 24.8 | 28.6 | 21.1 | 23.0 |
| *B. bronchiseptica* | OF |  |  |  |  |  |  |  |  |  |  |  |  |  |  |  |  |  |  |
| PCV3 | OF |  | 28.1 | 26.9 |  |  | 30.6 |  | 23.7 | 17.1 |  |  |  | 24.9 | 24.0 | 25.9 |  | 27.0 | 25.5 |
| PPV | OF | 21.2 | 14.0 | 23.8 |  | 22.8 | 24.4 | 20.2 | 23.6 | 18.3 | 26.5 | 19.2 | 26.2 | 13.6 | 19.5 | 20.0 | 22.2 | 22.8 | 22.4 |
| *M. hyopneumoniae* | OF |  |  |  |  |  |  |  |  |  | 22.6 |  | 24.5 |  |  |  |  |  |  |
| *M. hyorhinis* | OF |  |  |  |  |  |  |  |  |  |  |  |  |  |  |  |  |  |  |
| *P. multocida* | OF |  |  |  |  |  |  |  |  |  |  | 28.0 | 28.0 |  |  |  |  |  |  |
| *B. pilosicoli* | FS | 19.3 | 20.3 | 21.2 | 22.9 | 19.2 | 16.7 | 15.4 | 17.9 | 19.0 | 18.3 | 17.6 | 19.6 | 22.5 | 16.9 | 16.8 | 18.6 | 17.0 | 24.0 |
| *L. intracellularis* | FS |  | 17.1 |  |  |  |  | 16.4 |  |  |  |  |  | 19.3 | 19.5 |  |  |  |  |
| *E. coli* F4 | FS | 28.0 |  | 28.9 | 27.6 |  |  |  |  |  |  |  |  |  |  |  |  |  |  |
| *E. coli* F18 | FS |  |  |  |  |  |  |  |  | 25.2 |  |  |  | 28.9 |  | 28.2 |  |  |  |
| PCV3 | FS |  |  |  |  |  |  |  | 25.4 | 23.7 | 27.8 |  |  |  |  |  |  |  |  |
| PCV2 | FS | 16.6 | 16.8 | 15.9 | 17.1 | 20.8 | 21.3 | 13.9 | 12.8 | 16.0 | 20.0 | 19.6 | 17.8 | 10.7 | 10.2 | 19.8 | 20.0 | 22.9 | 18.8 |
| Rotavirus A | FS |  |  |  |  |  |  |  | 25.8 | 29.3 | 26.8 |  |  |  |  | 33.5 | 26.7 | 29.0 |  |
